# Microstructures and Properties of Al-Mg Alloys Manufactured by WAAM-CMT

**DOI:** 10.3390/ma15155460

**Published:** 2022-08-08

**Authors:** Yan Liu, Zhaozhen Liu, Guishen Zhou, Chunlin He, Jun Zhang

**Affiliations:** 1 Liaoning Provincial Key Laboratory of Advanced Material Preparation Technology, Shenyang University, Shenyang 110044, China; 2 School of Mechanical Engineering, Shenyang University, Shenyang 110044, China; 3 Liaoning Provincial Key Laboratory of Research and Application of Multiple Hard Films, Shenyang University, Shenyang 110044, China

**Keywords:** wire arc additive manufacturing, cold metal transfer, Al-Mg alloys, orthogonal experiment, microstructure, mechanical properties

## Abstract

A wire arc additive manufacturing system, based on cold metal transfer technology, was utilized to manufacture the Al-Mg alloy walls. ER5556 wire was used as the filler metal to deposit Al-Mg alloys layer by layer. Based on the orthogonal experiments, the process parameters of the welding current, welding speed and gas flow, as well as interlayer residence time, were adjusted to investigate the microstructure, phase composition and crystal orientation as well as material properties of Al-Mg alloyed additive. The results show that the grain size of Al-Mg alloyed additive becomes smaller with the decrease of welding current or increased welding speed. It is easier to obtain the additive parts with better grain uniformity with the increase of gas flow or interlayer residence time. The phase composition of Al-Mg alloyed additive consists of α-Al matrix and γ (Al_12_Mg_17_) phase. The eutectic reaction occurs during the additive manufacturing process, and the liquefying film is formed on the α-Al matrix and coated on the γ phase surface. The crystal grows preferentially along the <111> and <101> orientations. When the welding current is 90 A, the welding speed is 700 mm/min, the gas flow is 22.5 L/min and the interlayer residence time is 5 min, the Al-Mg alloy additive obtains the highest tensile strength. Under the optimal process parameters, the average grain size of Al-Mg alloyed additive is 25 μm, the transverse tensile strength reaches 382 MPa, the impact absorption energy is 26 J, and the corrosion current density is 3.485 × 10^−6^ A·cm^−2^. Both tensile and impact fracture modes of Al-Mg alloyed additive are ductile fractures. From the current view, the Al-Mg alloys manufactured by WAAM-CMT have a better performance than those produced by the traditional casting process.

## 1. Introduction

Additive manufacturing (AM) technology is a bottom-up layer-by-layer manufacturing method based on digital models [1,2], which has been widely utilized in consumer electronics, automotive engineering, aerospace as well as other industries [3]. Wire and arc additive manufacturing (WAAM) is a kind of AM technology that utilizes arc as the heat source and metal wire as a stacking material to manufacture components [4]. There are numerous branches of AM technology, which are selected according to the performance of the additive metal, the shape and size of the additive product, etc. Selective laser sintering (SLS) [5] and selective laser melting (SLM) [6,7,8,9] are generally used for complex components with high forming accuracy and small volume. For metal components with high forming environment requirements and a high energy absorption rate, electron beam freeform fabrication (EBF^3^) technology with wire feeding is generally adopted [10]. Compared with AM technologies such as SLS, SLM and EBF^3^, WAAM has the technological advantages of low equipment energy consumption, high material utilization rate and high deposition efficiency. It is suitable for additive manufacturing of large-sized components and has been widely used by researchers at home and abroad [11,12].

Aluminum alloys have low density, high strength, good electrical conductivity, thermal conductivity and corrosion resistance as well as machinability, and are widely utilized in aerospace and transportation as well as civil industry [13]. Al-Mg alloys belong to the 5XXX series of aluminum alloys. Compared with other aluminum alloys, the Mg element, as a crucial strengthening element, improves the strength and the corrosion resistance of Al-Mg alloys [14,15]. However, traditional aluminum alloy forming methods such as casting, forging and machining face challenges in meeting the current demand for intricate structural components [16,17]. Therefore, the use of WAAM technology to manufacture aluminum alloy components has become the focus of urgent research. Cold metal transfer (CMT) is a new melt inert gas welding technology [18,19]. In the welding process, the droplet of the welding wire makes contact with the molten puddle, a short circuit occurs, and the arc voltage is instantaneously close to zero. During the short circuit transition, the CMT welding technology can pull the droplet through the wire pulling back to overcome the surface tension of the droplet and avoid droplet rejection and necking burst [20,21]. Compared with other welding technologies using arc as the heat source, CMT welding technology has the characteristics of low heat input and no spatter as well as good forming quality, and has received extensive attention in recent years [22,23,24]. Cong B [25] utilized WAAM-CMT technology to manufacture Al-6.3%Cu alloys to investigate the application potential of aluminum alloys. The results showed that pores were produced when CMT additive was used to manufacture aluminum alloys, but the porosity defects were effectively eliminated by setting appropriate welding process parameters. Gu J [26] utilized the interlayer rolling technology to process 5087 aluminum alloys produced by the WAAM-CMT technology, which significantly reduced the number of aluminum alloyed pores and provided a solution to the problem of porosity defects during the welding process of the aluminum alloys. Geng H [27] utilized WAAM technology to manufacture 5A06 aluminum alloys and found that the tensile properties of the additive parts were anisotropic, with a difference of 22 MPa between transverse and longitudinal tensile strength. Zhang C [28] used variable polarity CMT as an additive heat source of Al-6Mg alloys, and the grain size of Al-6Mg was 20.6–28.5 μm. In addition to investigating the defects and microstructures of aluminum alloys manufactured by WAAM-CMT, it is also of great significance to investigate additive properties. Horgar A [29] utilized WAAM-CMT technology and AA5183 Al-Mg alloys welding wire as filling material, and the tensile strength of the sample was 293 MPa. Gu J [30] manufactured 5087 Al-Mg alloyed components with the WAAM-CMT process, and the average microhardness of the alloys was 107.2 HV. Su C [31] utilized the WAAM-CMT process to manufacture the Al-Mg alloys and found that the crystal size on the surface of the alloys was 42.9–88.7 μm, while that on the inside of the alloys was 37.7–77.6 μm. The average tensile strength of the sample was about 255 MPa.

At present, some progress has been made in the research of the WAAM-CMT process for additive manufacturing of Al-Mg alloyed materials [32,33,34]. However, there are still gaps in the related study of the WAAM-CMT process for the additive manufacturing of 5556 Al-Mg alloys. To further expand the application of Al-Mg alloys in the field of additive manufacturing, a WAAM-CMT system was established to manufacture Al-Mg alloyed additive walls. Based on the orthogonal experiments, the process parameters of the welding current (WC), welding speed (WS), gas flow (GF) and interlayer residence time (IRT) were adjusted to investigate the walking path and optimal process parameters of Al-Mg alloyed additive. The evolution law of microstructures and the effect of different process parameters on the mechanical properties of Al-Mg alloyed additive were analyzed.

## 2. Materials and Methods

The WAAM-CMT system consists of a TranspulsSynergic 3200 CMT welder manufactured by Fronius Austria and a computer numerical control (CNC) system. The additive manufacturing experiment was carried out on a 5052 Al-Mg alloyed substrate with 200 mm × 180 mm × 6 mm using ER5556 Al-Mg alloyed wire with a diameter of 1.2 mm as filler wire. The chemical composition of welding wire and substrate is shown in Table 1. During the experiment, the welding torch was controlled by the CNC system to complete the *X*, *Y* and *Z* axis movement on the welding platform, and pure Ar was used as the experimental protective gas. The dry elongation of the welding wire was 12 mm, the walking distance along the *X* direction was set as 150 mm, and the increment in the *Z* direction (the distance of the welding torch elevation after each deposition) was set as 2 mm. The number of layers of the additive was 20. Before the experiment, the substrate surface was wiped with acetone and it was ensured that the welding wire was dry. Combined with the purpose of the investigation, WC, WS, GF and IRT were taken as the orthogonal experimental variables to optimize the parameters. The orthogonal experiment table of L_25_(5^4^) was designed as shown in Table 2.

**Figure 1:**
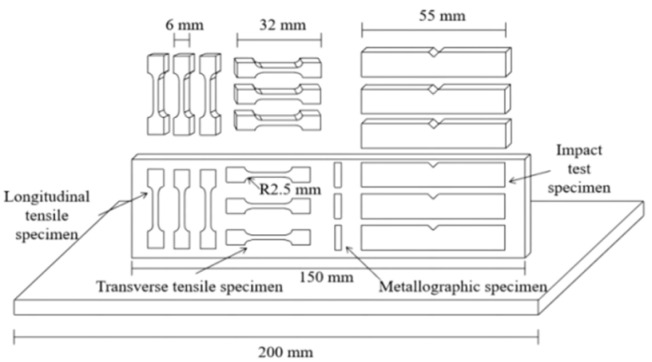
Schematic diagram of the sampling location and size.

The sampling location and size of the samples prepared for microstructure, tensile properties, and impact properties are shown in Figure 1. The microstructures of the Al-Mg alloyed wall (middle part) were observed. The sample was cut by a DK7763 super CNC wire-cutting machine according to the specified size, and the size of the metallographic piece was 10 mm × 2.5 mm × 1.5 mm. The size of the tensile specimen was determined according to GB/T 228.1-2021 “Metallic materials—Tensile testing—Part 1: Method of test at room temperature”. After grinding, electrolytic polishing was carried out in the DF-3010 electrolytic polishing corrosion tester with corrosion voltage controlled at 20 V, and the corrosion reagent was an alcohol solution of 10% perchloric acid. Three groups of tensile specimens were cut along the parallel and perpendicular to the processing direction, and the tensile strength was measured and averaged. The impact sample size was determined according to GB/T 229-2020 “Metallic materials—Charpy pendulum impact test—Part 1: Test method”. Three groups of impact samples were cut along the processing direction, and the impact absorption energy was measured and averaged.

A D/max-RB X-ray diffractometer (XRD) was utilized to analyze the phase composition of the additive. The scanning range was 5–90°. The microstructures of the Al-Mg alloyed components were analyzed by OLYMPUS-CK40M metallographic microscope (OM), S-4800 scanning electron microscope (SEM) and energy dispersive spectrometer (EDS). Electron back-scattered diffraction (EBSD) was used to obtain the orientation information of microscopic crystals in different process parameters and measure the average grain size. The testing equipment was S-4800 SEM with an EBSD probe. The data processing software of Channel 5 was used to calibrate the texture and crystal orientation of EBSD photos. At room temperature, the tensile test was carried out by a WDW-100B electronic universal testing machine at a tensile rate of 2 mm/min, and the impact test was carried out by the ZBC2602-C impact testing machine. The tensile and impact fracture morphologies were photographed by SEM. The PARSTAT-2273 electrochemical workstation was utilized to test the corrosion resistance of the sample by simulating seawater with a 3.5 wt% NaCl solution.

## 3. Results

### 3.1. Effect of the Walking Path on Al-Mg Alloyed Additive

**Figure 2:**
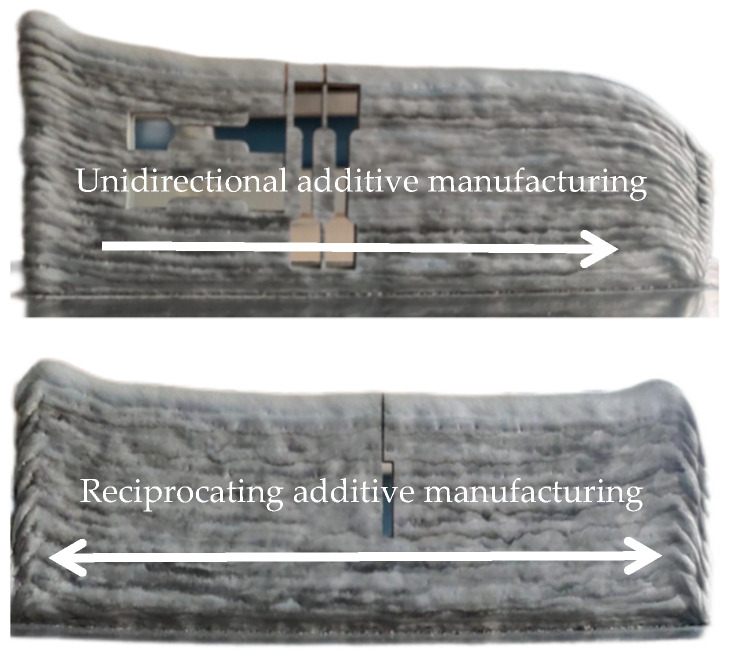
Al-Mg alloyed additive under different walking paths.

The Al-Mg alloyed additive under different walking paths is shown in Figure 2. It is observed that the forming effect of the Al-Mg alloyed additive walls under the reciprocating walking path is better than that under the unidirectional walking path. The additive parts collapse at the end of each layer under the unidirectional walking path. The unidirectional additive is a discontinuous process. At the end of each welding layer, the welding torch is raised and returned to the starting position to continue depositing the next layer of metal. In additive deposition, the initial part of the additive goes through the arc burning and arc extinguishing process of the CMT welding machine successively so that Al-Mg alloyed welding wire is melted and deposited on the surface of the substrate.

As the welding torch moves towards the specified deposition route, the arc burning and arc extinguishing processes are repeated, and the deposited Al-Mg alloys undergo the processes of heating and heat preservation as well as subcooling again. At the end of the additive, the metal is no longer subjected to an intricate thermal cycle as the welding torch discontinues moving forward because of arc extinction. Eventually, as the number of additive layers increases, collapse occurs at the end of unidirectional addition. It is observed that the WAAM walking path has a particular influence on the geometry of the additive. The continuous reciprocating way makes the thermal cycle at the beginning or end of the additive highly symmetrical but also improves the wetting effect during the additive process. Therefore, the Al-Mg alloyed additive manufactured by WAAM-CMT using the reciprocating walking path has a continuous forming, good appearance and no obvious welding defects, which avoids the problem of collapse in the additive process.

### 3.2. Optimal Process Parameters Based on Orthogonal Experiment

Based on the reciprocating additive manufacturing walking path, the transverse tensile strength (TTS) of Al-Mg alloyed additive components were measured under different process parameters in the orthogonal experiment. The results are shown in Table 3. The direction of transverse tensile strength is the walking direction of the welding torch. The effect of thermal cycling conditions on the alloys is the same in this direction, so the microstructural distribution of the alloys is more uniform. The TTS is finally taken as the criterion to evaluate the mechanical properties of the orthogonal experiments.

K_i_ is the average value of all TTS results of this factor at the level of i, and R is the range of K_i_. In other words, R = k_max_ − k_min_. The larger k_i_ indicates the optimal experimental results at the i level. The larger R is, the greater the influence of this factor on the experimental results is. According to the range analysis results, the optimal process parameters at the highest TTS are as follows: WC = 90 A, WS = 700 mm/min, GF = 22.5 L/min, IRT = 5 min. At this time, the TTS of Al-Mg alloyed additive can reach 382 MPa. Under the experimental parameters, WC is the most crucial factor affecting the TTS of Al-Mg alloyed additive.

## 4. Discussion

### 4.1. Pore Analysis of Al-Mg Alloyed Additive under Orthogonal Experiment

The metallographic microstructures and porosity of Al-Mg alloyed walls are shown in Table 4. The black granular material is a porosity defect formed during the additive process. Due to the good thermal conductivity of aluminum alloys, the molten puddle cooling speed is extremely fast. The escape time of bubbles in the molten puddle is not sufficiently fast, and the molten puddle begins to crystallize and solidify, thus forming the porosity defects. According to Li Z et al. [35], the pores formed in the aluminum alloyed additive process are especially hydrogen pores. The hydrogen pores mainly generate heterogeneous nucleated particles at grain boundaries and grow up through free diffusion and merger. The results demonstrate a competitive growth relationship between the formation of pores and the crystal structure. When the heat input of the additive is smaller, the grain size of the alloys becomes more minor, and the size and number of pores generated are smaller. On the contrary, the larger the additive heat input, the larger the number and size of pores.

It can be seen from Table 4 that the formation of porosity is closely related to the heat input during the additive process. Porosity is the total area of the pore divided by that of metallography. To ensure the accuracy of the experiment, the average value of each parameter sample was taken from three groups. To improve the tensile strength of the alloy, the porosity must be reduced. The porosity is the highest under the experimental parameters of 16# (WC = 120 A, WS = 400 mm/min, GF = 20 L/min, IRT = 5 min), and the higher porosity results in a sharp decrease in the TTS under 16# orthogonal parameters. At the same time, it is observed that under the practical parameters of 5# (WC = 90 A, WS = 800 mm/min, GF = 22.5 L/min, IRT = 5 min), the porosity is the lower, and the corresponding TTS is the highest. The reason is that under the experimental parameters of 5#, the welding current is small, and the welding speed is considerable, resulting in low heat input in the depositing process. On the one hand, the lower the welding heat input, the less the elements evaporated due to overheating during the additive process, and the fewer bubbles were generated in the molten puddle, which is the main factor in avoiding the formation of pores. On the other hand, the smaller the welding heat input, the shorter the residence time of the molten puddle at a high temperature, and the more sufficient the escape time of tiny bubbles in the molten puddle was, to reduce the porosity and give the Al-Mg alloyed additive better mechanical properties. In addition, compared with the experimental parameters of 16#, the experimental parameters of 5# have a faster gas flow protection to isolate the gas in the outside air from penetrating the Al-Mg alloys and strengthen the security of the alloyed structure. Therefore, under the WAAM-CMT process, decreasing the welding current or increasing the welding speed is to decrease the heat input. WAAM-CMT technology can effectively reduce or even eliminate pore defects due to the reduction of heat input, thus enhancing the mechanical properties of Al-Mg alloyed additives.

### 4.2. Grain Size Analysis of Al-Mg Alloyed Additive under Different Process Parameters

**Figure 3:**
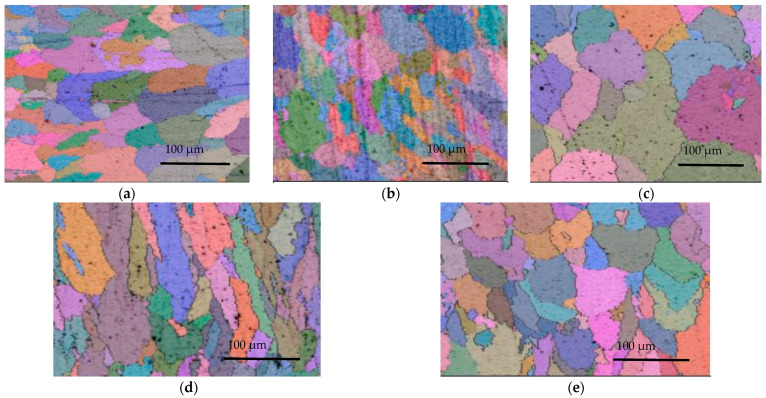
EBSD microstructure of Al-Mg alloyed additive under different process parameters: (**a**) Contrasting sample (90 A, 800 mm/min, 22.5 L/min, 5 min); (**b**) 130 A welding current; (**c**) 400 mm/min welding speed; (**d**) 12.5 L/min gas flow; (**e**) 1 min interlayer residence time.

The influence of different process parameters on the EBSD microstructure of Al-Mg alloyed additive was studied by single-factor analysis, and the test results are shown in Figure 3. The grains with different grain orientations were marked with different colors by EBSD to analyze the grain sizes. Figure 3a shows the EBSD microstructure of Al-Mg alloyed additive manufactured under the process parameters of WC = 90 A, WS = 800 mm/min, GF = 22.5 L/min as well as IRT = 5 min. Taking Figure 3a as the contrasting sample, the process conditions in Figure 3b–e only change the welding current, welding speed and gas flow, as well as interlayer residence time, while keeping other process parameters unchanged.

Figure 3b shows the EBSD microstructure of the additive under the welding current of 130 A. Compared with Figure 3a (90 A), the microstructures of the additive become coarser with the increase of welding current. The reason is that the growth of welding current increases the heat input in the WAAM-CMT process. The rise in the heat input increases the heat dissipation time of the additive, and the grain has sufficient time to grow. Therefore, the grain size is relatively coarse when the welding current is 130 A. Figure 3c shows the EBSD microstructure of additive at a welding speed of 400 mm/min. In the WAAM-CMT process, welding speed is also one of the crucial factors affecting the heat input of additive. Compared with Figure 3a (800 mm/min), when the welding speed is slower, the volume of arc melting wire per unit length is more extensive, resulting in increased additive heat input and grain coarsening of the Al-Mg alloyed additive. Therefore, decreasing welding current or increasing welding speed can reduce the heat input of the welding process, refine grain size and form an equiaxed grain structure.

Figure 3d shows the EBSD microstructure of the additive at a gas flow rate of 12.5 L/min. Compared with Figure 3a (22.5 L/min), the grains are columnar, and the size distribution is uneven when the gas flow rate is slow. The reason is that the shielding gas plays a role in isolating the outside air and can also play a role in optimizing the weld bead. When the gas flow is languid, the weld bead is weakened by the gas pressure, causing the grains to grow outward in the form of columnar crystals. Figure 3e shows the EBSD microstructure of the additive under the interlayer residence time of 1 min. Compared with Figure 3a (5 min), it is found that the microstructural microstructure obtained under different interlayer residence time is good. Still, when the interlayer residence time is 5 min, the grain size distribution of the additive is more uniform. The reason is that interlayer residence time plays an essential role in improving the thermal accumulation effect of additive parts. The shorter the residence time between layers, the less heat accumulated in each layer is lost. The accumulated heat will prolong the holding time of grains, and the grains of Al-Mg alloyed additive have sufficient conditions to grow. As the number of additive layers increases, the heat accumulation effect becomes more evident. Therefore, increasing gas flow rate and prolonging interlayer residence time is beneficial for obtaining the grains with good uniformity.

**Figure 4:**
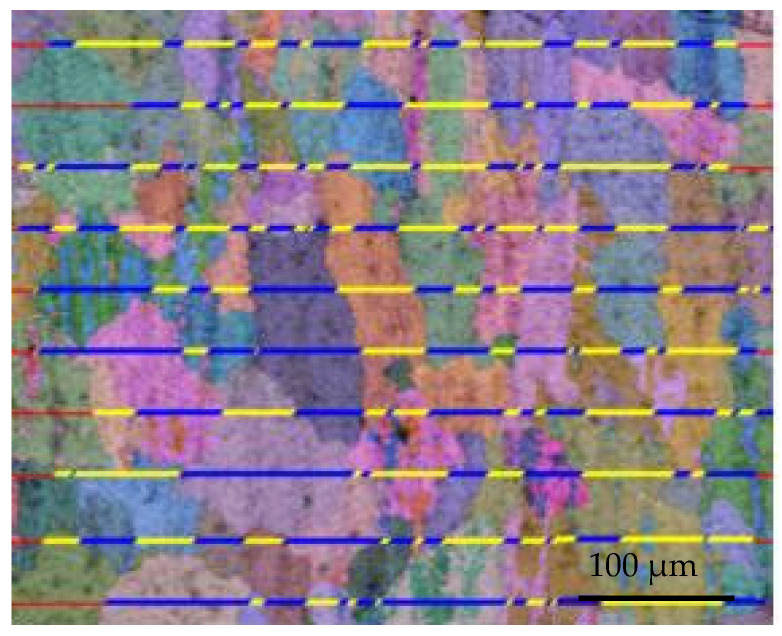
Grain size of Al-Mg alloyed additive measured by scribing method.

In the WAAM-CMT process, the additive heat input is the fundamental factor affecting the crystal size of the additive, and the additive heat input is mainly affected by the welding current and welding speed. As the welding current decreases from 130 A to 90 A, or as the welding speed increases from 400 mm/min to 800 mm/min, the grain size of Al-Mg alloys becomes smaller. The results of the EBSD grain size analysis demonstrate that the microstructures of Al-Mg alloyed additive are mainly equiaxed grain and columnar grain. Due to the existence of columnar crystals, the Al-Mg alloyed additive is anisotropic. As the gas flow rate increases from 12.5 L/min to 22.5 L/min, the equiaxed grains are easily obtained. The reduction of columnar crystals gives the material better microstructures and properties. With the interlayer residence time increasing from 1 min to 5 min, it is easier to obtain Al-Mg alloyed additive with good microstructural uniformity and fine grain size. The results of orthogonal tensile experiments demonstrate that the optimal tensile strength of Al-Mg alloys is brought under the conditions of 90 A welding current, 700 mm/min welding speed, 22.5 L/min gas flow rate and 5 min interlayer residence time. The average grain size of the additive is 25 μm under the optimal process parameters measured by the transverse scribing method in Channel 5 software, and the measurement method is shown in Figure 4.

### 4.3. Phase Analysis of Al-Mg Alloyed Additive

**Figure 5:**
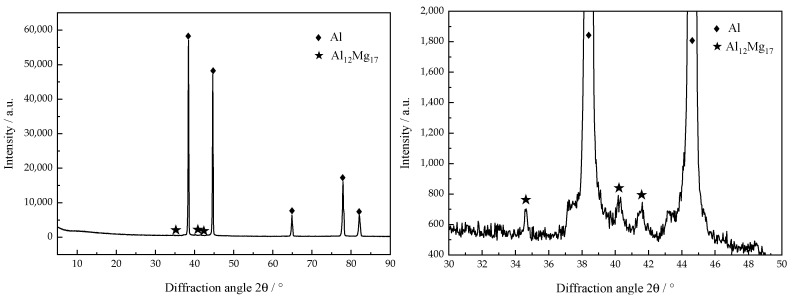
XRD pattern of Al-Mg alloyed additive.

The XRD pattern of the Al-Mg alloyed additive is shown in Figure 5. It can be seen that there are only diffraction peaks of the α-Al phase and a small amount of Al_12_Mg_17_ phase in the XRD pattern of the sample. Due to the relatively small content of other elements, they do not appear in the XRD pattern. Under the action of the CMT welding heat source, Al-Mg alloyed welding wire melts to form a molten puddle. As the CMT welding technology has the characteristics of short-circuit transition, the decrease of arc and droplet temperature leads to the extremely short existence of the molten puddle. The molten puddle, after supercooling, will crystallize and precipitate on the substrate or the previous Al-Mg alloyed layer. When the temperature reaches between eutectic temperature and liquidus temperature, the material will undergo the eutectic reaction: L ⇌ Mg + γ (Al_12_Mg_17_).

Although the heat accumulated by the last metal layer on the previous metal layer is increasing, the area of the molten puddle cooling to the surrounding environment is also increasing. When the number of layers increases to a certain height, the sediments stay in thermal equilibrium. The heat accumulation in the WAAM-CMT process is the main reason for the microstructural transformation. Under the heat accumulation effect, the preheating effect of the former layer of metal on the last layer of metal is gradually strengthened, which leads to the extension of the holding time for the growth of various microstructures in Al-Mg alloys, thus forming larger grains. With the increase of additive layers, the heat absorption and dissipation of the molten puddle reaches a balanced state in unit time, and the heat accumulation effect gradually tends to be gentle. Therefore, the interception position of the metallographic sample is the 10th layer of the additive wall.

**Figure 6:**
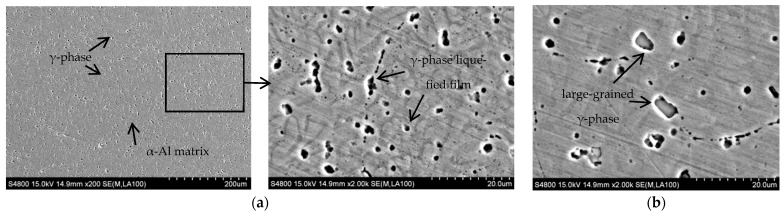
SEM microstructure of Al-Mg alloyed additive under different linear energies: (**a**) 1440 J/cm line energy; (**b**) 1800 J/cm line energy.

Line energy is an essential means to characterize heat input. The calculation formula of line energy is *q* = *UI*/*V*, where *U* is the welding voltage, *I* is the welding current, and *V* is the welding speed. Under WC = 90 A, GF = 20 L/min as well as IRT = 2 min, the SEM microstructure of Al-Mg alloyed additive with different line energy is shown in Figure 6.

**Figure 7:**
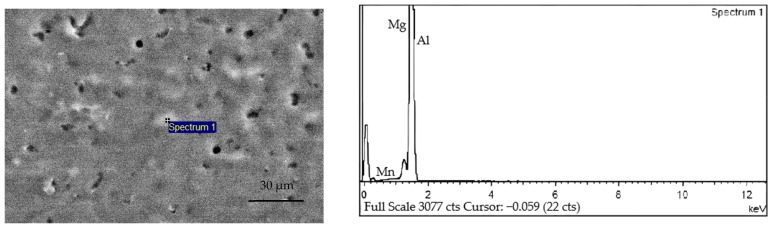
EDS point scanning results of Al-Mg alloyed additive.

Figure 6a shows the SEM microstructure of the additive at different magnifications under the condition of 1440 J/cm line energy. It is observed that the α-Al phase is the alloyed matrix, and there is a region of liquefaction. In addition, the eutectic reaction occurs to form the Mg and γ phase. When the next layer of metal is deposited, the upper layer of metal is liquefied again by the Mg and γ phase due to heating then redissolved into the molten α-Al matrix. However, due to the extremely short time of the CMT welding heat source, the solid-state phase transformation of the γ phase is minimal, so the γ phase does not have enough time to dissolve into the α-Al matrix fully. When the temperature is reduced to the eutectic reaction temperature, the Mg and γ phase will crystallize again, forming the eutectic liquid phase. As the eutectic reaction time is extremely short, the liquefied film is formed at the part of the original Mg and γ phase as the temperature continues to decrease. Figure 6b shows the SEM microstructure of the additive under the condition of 1800 J/cm line energy. Compared with 1440 J/cm line energy, with the increase of heat input, the nucleation of γ phase crystal is not uniform, accompanied by the appearance of a large-grained γ phase microstructure. To determine the composition of black particles in the SEM microstructure of Al-Mg alloyed additive, EDS point scanning was performed for the black particles, as shown in Figure 7. EDS point scanning results demonstrate that Al and Mg are the main elements in the particles. Combined with the XRD pattern and analysis results, it is verified that the black substance is the γ phase microstructure. The microstructures are improved by reducing the heat input, thereby increasing the mechanical properties of the Al-Mg alloyed additive. On the one hand, reducing the heat input can avoid the formation of the γ phase with large grains. On the other hand, the decrease of heat input makes the grains fine and uniform, and the dislocation movement is hindered by the effect of fine-grain strengthening to improve the mechanical properties of Al-Mg alloyed additive.

The chemical composition of the additive is shown in Table 5. The results show that the Mg element content decreases from 4.9% to 4.68%, and the loss rate is 4.49%. Yuan, T [36] fabricated the Al-Mg alloy by the WAAM-TIG technology and studied the loss of the Mg element under different process parameters. The results show that the loss rate of the Mg element is 5.56% at the optimum process parameters. Because the WAAM-CMT has the significant advantage of the short circuit transition, the loss of Mg element in the Al-Mg alloy is slightly reduced compared with the WAAM-TIG.

### 4.4. Crystal Orientation Analysis of Al-Mg Alloyed Additive

**Figure 8:**
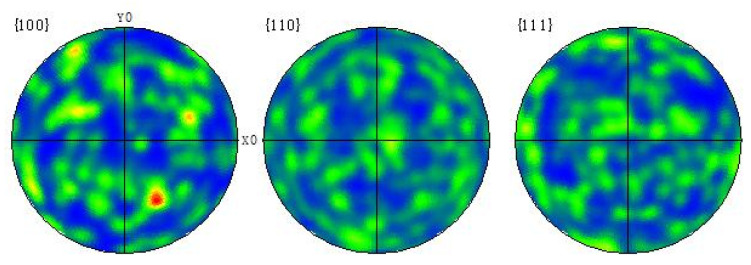
Polar diagrams of Al-Mg alloyed additive on different planes.

Using EBSD crystal orientation imaging technology to obtain crystal orientation measurement data is beneficial for analyzing the change of texture in the sample. Figure 8 is the polar diagram of Al-Mg alloyed additive on different planes when the TTS is optimal. It can be seen that there is a <111> texture on the XY plane at 45° off the *X* direction, while there is no preferred orientation on other crystal planes. The results indicate that Al-Mg alloyed additive grows preferentially in the <111> orientation, which is consistent with the close-packed plane of α-Al with the face-centered cubic crystal structure.

**Figure 9:**
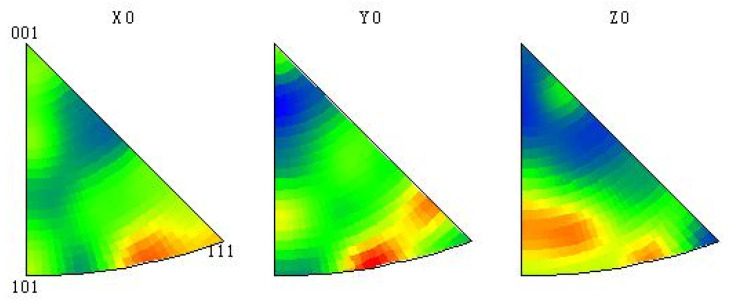
Inverse pole diagrams of Al-Mg alloyed additive on different planes.

**Figure 10:**
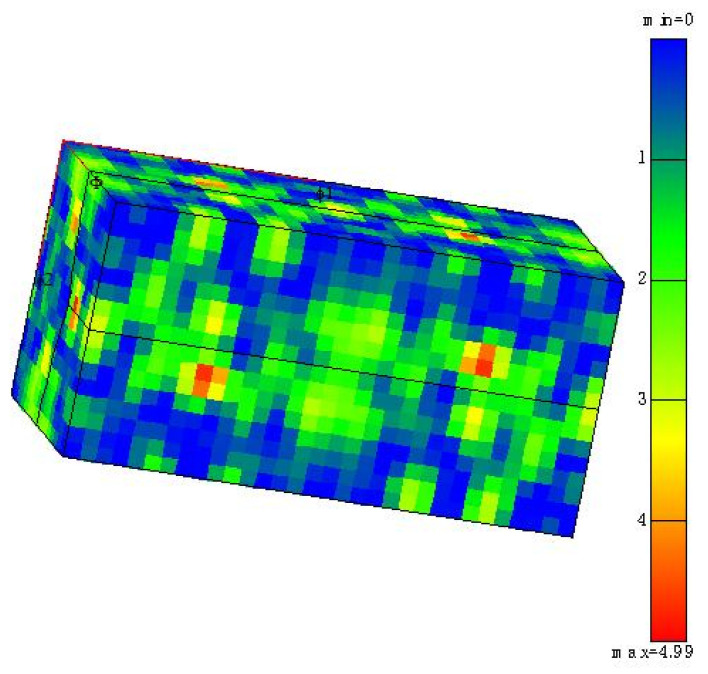
Orientation distribution diagram of Al-Mg alloyed additive.

To further verify the rationality of the analysis, the samples were processed with an inverse pole diagram as shown in Figure 9. By calibrating the crystal coordinates with the samples, it is found that the texture of the sample exists not only in the <111> orientation but also in the <101> orientation. However, the crystal orientation intensity of the <101> is less than that of the <111>. The <101> plane is the close-packed plane of the γ phase with the body-centered cubic structure, indicating that the crystal grows preferentially along the <111> plane during the additive process. On the contrary, if the γ phase is preferentially extended, the liquefied film will not form at the part of the Mg and γ phase. Therefore, in the process of manufacturing Al-Mg alloyed additive with WAAM-CMT technology, the crystals grow preferentially along the <111> and <101> orientations. In contrast, the crystals which deviate significantly from the <111> and <101> orientations will discontinue growing. The crystal orientation distribution of the Al-Mg alloyed additive is shown in Figure 10. In the aluminum alloyed matrix, Mg mainly grows along with the <101> orientation and forms the γ phase, resulting in lattice distortion, which significantly strengthens the hinderance of dislocation movement and has a solid solution strengthening effect on the alloys.

### 4.5. Performance Analysis of Al-Mg Alloyed Additive

#### 4.5.1. Analysis of Tensile Properties and Fracture Morphology

**Figure 11:**
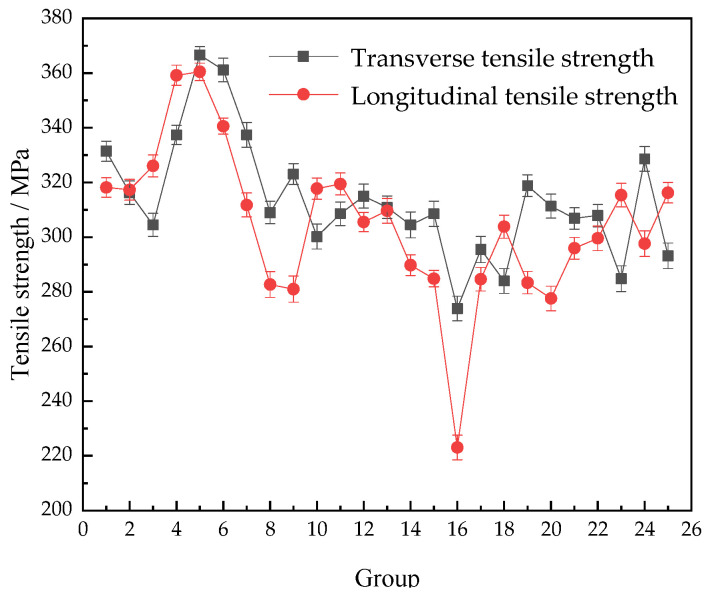
Tensile strength of Al-Mg alloyed additive under different process parameters.

The transverse and longitudinal tensile strengths of the Al-Mg alloyed additive measured under different process parameters of the orthogonal experiment in Table 3 above are shown in Figure 11.

There is little difference between the transverse and longitudinal tensile strengths of the Al-Mg alloyed additive on the whole. Still, the transverse tensile strength of the additive is slightly higher than the longitudinal tensile strength. The reason is that in the process of the additive, each layer of metal in the transverse direction is heated to melt and supercooled to crystallize, forming a uniform and stable Al-Mg alloyed microstructure. In the longitudinal order, the problem of interlayer microstructural combination needs to be considered. Due to the longitudinal gradient diffusion of heat centered on the welding torch and the gravity effect of liquid metal, the heating cycle and heat accumulation conditions of the lower metal and the upper metal are different, so the longitudinal interlayer microstructure combination is worse than the transverse interlayer microstructure combination.

**Figure 12:**
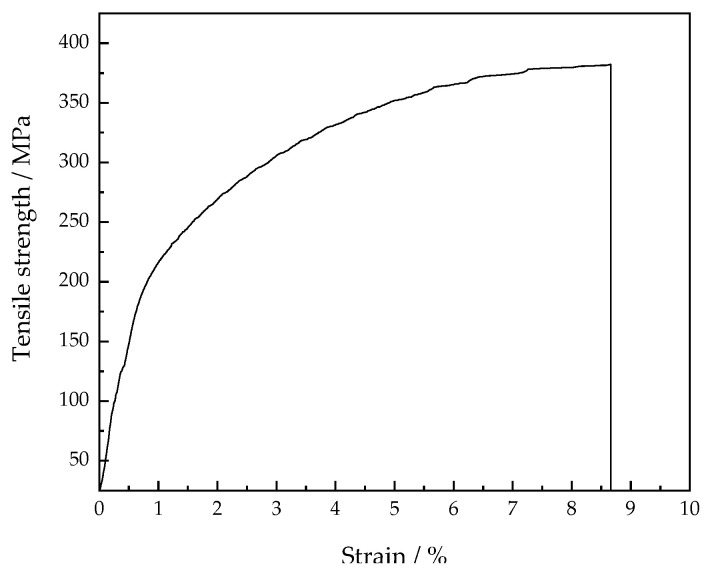
The tensile stress-strain curve of the optimal parameters.

The microstructures of the Al-Mg alloyed additives manufactured by WAAM-CMT technology are anisotropic, but the effect is not obvious. The average tensile strength of the Al-Mg alloyed additives is 310 MPa, which is approximately 30% higher than that of the matrix. Traditional Al-Mg alloys are manufactured by stabilizing annealing or work hardening process, and the tensile strength is generally between 200 MPa and 400 MPa [37]. The optimum tensile strength of the Al-Mg alloyed additive manufactured by WAAM-CMT technology is 382 MPa, which is higher than that of the annealed Al-Mg alloys and some machined Al-Mg alloyed forgings. The tensile stress–strain curve of the optimal parameters is shown in Figure 12. The experimental results can provide the experimental basis and data reference for the manufacturing process of Al-Mg alloys by WAAM-CMT.

**Figure 13:**
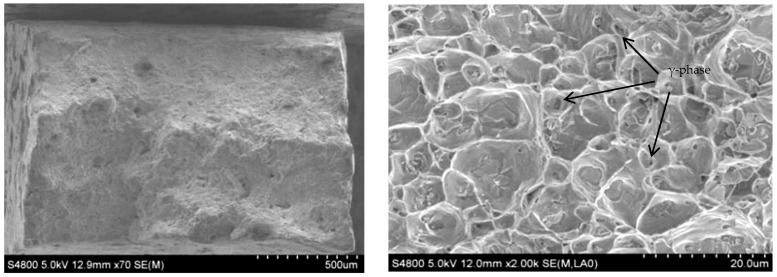
SEM morphology of transverse tensile fracture of Al-Mg alloyed additive.

The SEM morphology of transverse tensile fracture of the additive is shown in Figure 13. It can be seen that the cross-sectional area at the rupture of the tensile specimen shrinks, and the necking phenomenon occurs. It demonstrates that during resisting tensile deformation, dislocations are constantly produced inside the material, and the dislocations at grain boundaries or γ phase particles are used to fight the tensile deformation caused by the outside of the material. The fracture surface of the sample is gray–white, with a cup-cone shape around it and a tearing edge. A large number of dimples were observed in the fracture under a high-power microscope. Therefore, the fracture mode of the Al-Mg alloyed additive is a ductile fracture. In general, the formation of the dimple of Al-Mg alloys is related to the precipitation of the γ phase, and the size, microstructure and distribution of the γ phase affect the fracture mode of the material.

#### 4.5.2. Analysis of Impact Properties and Fracture Morphology

The impact property is also a crucial mechanical property index for Al-Mg alloyed additive according to the different service environments of materials. Under WC = 90 A, WS = 700 mm/min, GF = 22.5 L/min and IRT = 5 min, the average impact absorption energy of Al-Mg alloyed additive measured at room temperature is 26.19 J, which is higher than the standard value of 24.00 J of Al-Mg alloys. Yan X [38] adopted the plasma arc welding process to weld the 5052 Al-Mg alloyed substrates with ER5356 Al-Mg alloyed welding wire, and the average impact absorption energy was 19.60 J. Compared with this process, the impact property of Al-Mg alloyed additive manufactured by WAAM-CMT technology is improved by 22%.

**Figure 14:**
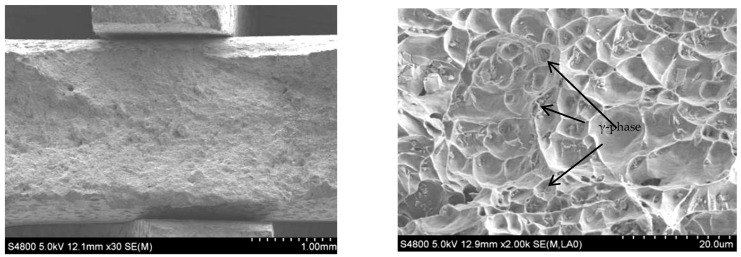
SEM morphology of impact fracture of Al-Mg alloyed additive.

The impact fracture morphology of the additive is shown in Figure 14. There are many dimples at the impact fracture, which indicates that the fracture mode is ductile fracture. The Al-Mg alloy grains manufactured by WAAM-CMT are mainly equiaxed. Once subjected to the impact load, the grains will produce plastic deformation along the shear slip direction. Compared with the SEM microstructure of the Al-Mg alloyed additive in Figure 6 above, the grains are elongated to produce the small-angle grain boundaries under the action of shear stress. The large-angle grain boundaries are generated from the small-angle grain boundaries with plastic deformation, which is finally manifested as the microscopic morphology in Figure 14. The sediments formed at grain boundaries become the core of grain recrystallization and promote the impact deformation resistance of microstructures. Under the impact load, the dislocation cannot resist the impact load, and fracture failure occurs at the weak position, such as the grain boundary, before it has sufficient time to accumulate to the grain boundary or γ phase fully. In conclusion, Al-Mg alloyed additive manufactured by WAAM-CMT technology has excellent mechanical properties and can meet the basic requirements of modern production and manufacturing.

#### 4.5.3. Corrosion Resistance Analysis

**Figure 15:**
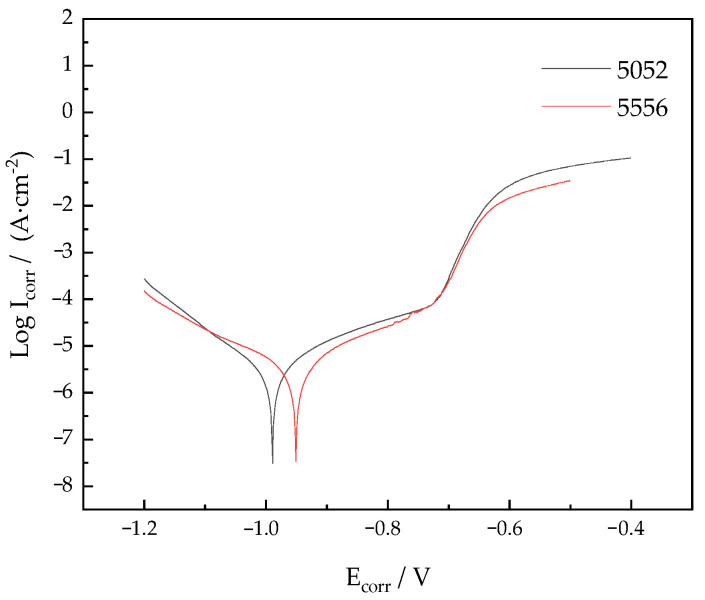
Polarization curves of Al-Mg alloys.

Electrochemical tests were carried out on 5556 Al-Mg alloyed additive and 5052 Al-Mg alloyed experimental substrates, and the polarization curves were measured as shown in Figure 15. It is observed that the corrosion current density (I_corr_) of Al-Mg alloyed additive is 3.485 × 10^−6^ A·cm^−^^2^, while that of Al-Mg experimental substrate is 4.685 × 10^−6^ A·cm^−^^2^. The corrosion current density of Al-Mg experimental substrate is slightly higher than that of the additive. The higher the corrosion current density, the faster the corrosion rate of the material. In addition, the corrosion potential (E_corr_) of the 5556 additive is −0.95 V, while that of the substrate is −0.99 V. The more positive the corrosion potential, the stronger the corrosion inertness of the material. Therefore, the additive provides better corrosion resistance than the substrate. Compared with the α-Al matrix, the γ phase is the anode, and the corrosion reaction occurs preferentially. Therefore, the alloys have a great sensitivity to intergranular corrosion.

References [39,40] show that the properties of the Al-Mg alloy are improved with the increase of the Mg element content. However, when the content of the Mg element exceeds 7%, the precipitates of the Mg element increase and the crystallization hot crack occurs. In the study, the Mg content of the 5556 Al-Mg additive manufactured by WAAM-CMT is 4.68%, while the Mg content of the 5052 Al-Mg substrate is 2.54%. Since the Mg content of the 5556 Al-Mg additive is higher than that of the 5052 Al-Mg alloy, the γ phase with a relatively large volume fraction acts as a corrosion barrier to inhibit the corrosion of the matrix. Therefore, the 5556 Al-Mg alloy additive has better corrosion resistance. From the current view, the 5052 Al-Mg alloy is one of the most commonly used Al-Mg alloys. Because of its good corrosion resistance, it is widely utilized in the manufacturing of aircraft fuel tanks and ship sheet metal components. According to the polarization curve data, the corrosion resistance of 5556 Al-Mg alloyed additive is 1.34 times that of 5052 Al-Mg alloyed substrate. Therefore, 5556 Al-Mg alloyed additives can be operated in a particular seawater corrosion environment.

## 5. Conclusions

(1) For Al-Mg alloyed additive manufactured by WAAM-CMT, the forming effect of the reciprocating walking path is better than that of the unidirectional walking path. Based on the orthogonal experiment, the optimal process parameters for the highest tensile strength are as follows: 90 A of welding current, 700 mm/min of welding speed, 22.5 L/min of gas flow and 5 min of interlayer residence time;

(2) As the welding current decreases from 130 A to 90 A, the welding speed increases from 400 mm/min to 800 mm/min, the gas flow increases from 12.5 L/min to 22.5 L/min, the interlayer residence time increases from 1 min to 5 min, and the grain size of Al-Mg alloyed additive is gradually refined. The microstructures are mainly equiaxed and columnar crystals. Under the optimal process parameters, the average grain size of Al-Mg alloyed additive is 25 μm;

(3) Al-Mg alloyed additive is mainly composed of the α-Al phase and a small amount of Al_12_Mg_17_ phase, and the eutectic reaction will occur in the additive process: L ⇌ Mg + γ (Al_12_Mg_17_). Mg exists in the form of Al_12_Mg_17_ solid solution in Al-Mg alloyed additive. With the alternation of the thermal cycle, the eutectic reaction results in liquefied film at the original Mg and γ phase locations. The larger the welding heat input, the larger the grain size of the liquid film. During the process of WAAM-CMT manufacturing, the crystals of Al-Mg alloyed additive mainly grow preferentially along the orientations of <111> and <101>. At the same time, the preferred strength of <111> is greater than that of <101>;

(4) The maximum tensile strength of Al-Mg alloyed additive is 382 MPa, the average tensile strength is 310 MPa, and the average impact absorption energy is 26.19 J. Under static tensile load or impact load, ductile fracture occurs in all Al-Mg alloyed additive parts, and there are many dimples in the fracture. The corrosion resistance of the Al-Mg alloyed additive is better than that of the substrate.

## Figures and Tables

**Table 1:** Chemical composition of the substrate and filler material (mass fraction/%).

Materials	Si	Fe	Cu	Mn	Mg	Cr	Zn	Ti	Al
5052	0.25	0.40	0.10	0.10	2.2–2.8	0.15–0.35	0.10	0.01	Bal.
ER5556	0.07	0.17	0.01	0.61	4.90	0.11	0.08	0.09	Bal.

**Table 2:** Range of matrix building parameters with four factors and five levels.

Factors	Levels				
	1	2	3	4	5
WC/A	90	100	110	120	130
WS/mm·min^−1^	400	500	600	700	800
GF/L·min^−1^	12.5	15.0	17.5	20.0	22.5
IRT/min	1	2	3	4	5

**Table 3:** Experimental results of transverse tensile strength under orthogonal process parameters.

No.	WC/A	WS/mm·min^−1^	GF/L·min^−1^	IRT/min	TTS/MPa	No.	WC/A	WS/mm·min^−1^	GF/L·min^−1^	IRT/min	TTS/MPa
1	90	400	12.5	1	331	17	120	500	22.5	3	296
2	90	500	15.0	2	316	18	120	600	12.5	4	284
3	90	600	17.5	3	304	19	120	700	15.0	5	319
4	90	700	20.0	4	337	20	120	800	17.5	1	311
5	90	800	22.5	5	367	21	130	400	22.5	4	307
6	100	400	15.0	3	361	22	130	500	12.5	5	308
7	100	500	17.5	4	337	23	130	600	15.0	1	285
8	100	600	20.0	5	309	24	130	700	17.5	2	329
9	100	700	22.5	1	323	25	130	800	20.0	3	293
10	100	800	12.5	2	300						
11	110	400	17.5	5	309	k_1_	331.24	316.37	305.60	310.30	
12	110	500	20.0	1	315	k_2_	326.15	314.40	310.56	310.56	
13	110	600	22.5	2	311	k_3_	309.49	298.63	299.57	299.57	
14	110	700	12.5	3	304	k_4_	296.71	322.48	305.68	319.57	
15	110	800	15.0	4	309	k_5_	304.27	315.98	320.60	327.86	
16	120	400	20.0	2	274	R	34.53	23.85	21.03	28.29	

**Table 4:** Al-Mg metallographic structure under orthogonal process parameters.

Al-Mg Metallographic Structure and Porosity under Orthogonal Experiment ^1^ 200 Μm				
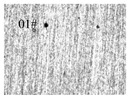	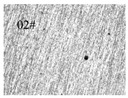	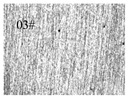	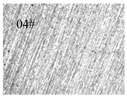	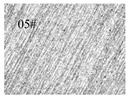
0.7%	1.6%	1.0%	0.7%	0.5%
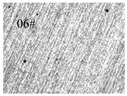	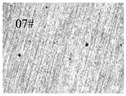	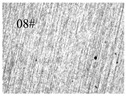	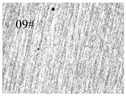	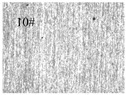
1.9%	1.9%	0.9%	0.8%	1.3%
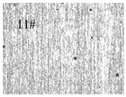	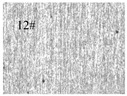	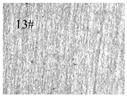	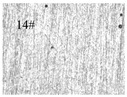	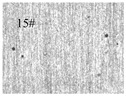
2.0%	1.2%	0.8%	2.0%	1.7%
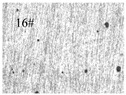	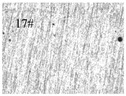	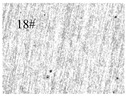	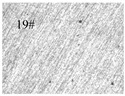	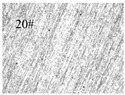
5.4%	1.8%	1.3%	1.1%	1.1%
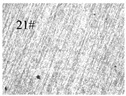	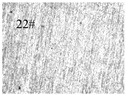	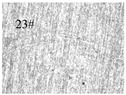	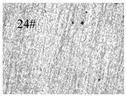	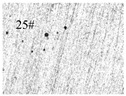
2.0%	0.5%	0.4%	0.8%	3.0%

^1^ The specific values of 01#–25# orthogonal process parameters correspond to Table 3 above.

**Table 5:** Chemical composition of the substrate and additive material (mass fraction/%).

Materials	Si	Fe	Cu	Mn	Mg	Cr	Zn	Ti	Al
5052	0.25	0.39	0.11	0.10	2.54	0.21	0.09	0.01	Bal.
5556	0.08	0.18	0.01	0.63	4.68	0.12	0.07	0.10	Bal.

## Data Availability

Not applicable.
